# Supplementary material for: Effectiveness of the ALT/AST ratio for predicting insulin resistance in a Korean population: A large-scale, cross-sectional cohort study
Source: PLoS One. 2024 May 17;19(5):e0303333. doi: 10.1371/journal.pone.0303333 (PMC11101110; doi:10.1371/journal.pone.0303333)
Supplement: S1 Fig — Distributions of ALT, AST, ALT/AST in Korean Men (A) and women. (DOCX) [file pone.0303333.s001.docx]

**Effectiveness of the ALT/AST ratio for predicting insulin resistance in a Korean population: A large-scale, cross-sectional cohort study (Supplementary Materials)**

Seul Ki Han^1,3¶^, Myung Jae Seo^2, 3¶^, Taesic Lee^2, 3, 4^, Moon Young Kim^1,3*^

^1^ Division of Gastroenterology and Hepatology, Department of Internal Medicine, Yonsei University Wonju College of Medicine, Wonju, Korea

^2^ Department of Family Medicine, Yonsei University Wonju College of Medicine, Wonju, Korea

^3^ Regeneration Medicine Research Center, Yonsei University Wonju College of Medicine, Wonju, Korea

^4^ Division of Data Mining and Computational Biology, Institute of Global Health Care and Development, Wonju, Korea

**Supplementary Figure 1**. Distributions of ALT, AST, ALT/AST in Korean Men (A) and women.

| (A)  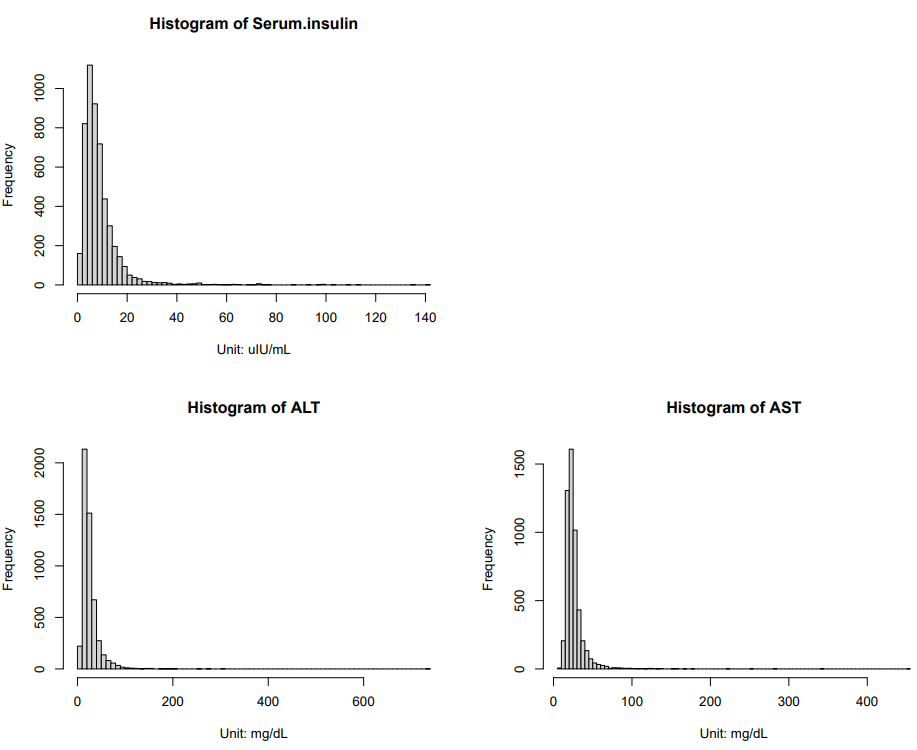 |
| --- |

| (B)  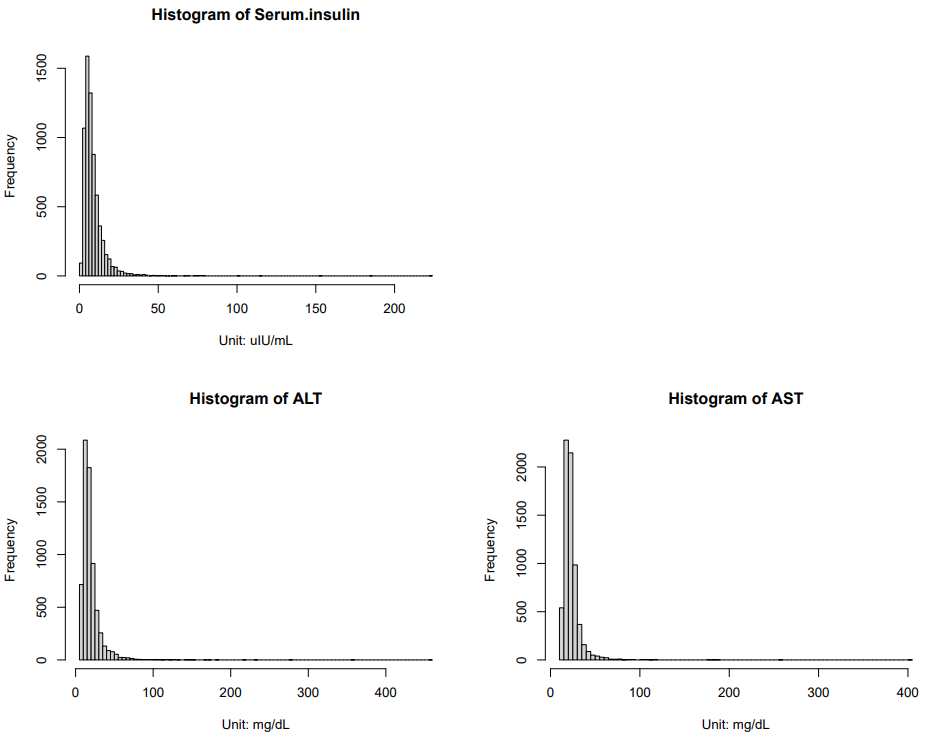 |
| --- |
