# Supplementary material for: Effectiveness of the ALT/AST ratio for predicting insulin resistance in a Korean population: A large-scale, cross-sectional cohort study
Source: PLoS One. 2024 May 17;19(5):e0303333. doi: 10.1371/journal.pone.0303333 (PMC11101110; doi:10.1371/journal.pone.0303333)
Supplement: S1 File — (ZIP) [file pone.0303333.s002.zip › PLOS Human Participants Research Checklist.docx]

**Human Participants Research Checklist**

***Complete the following if your study involved human participants or human participants’ data. These questions should be addressed for prospective and retrospective studies.***

1. Did you obtain ethics approval for this study?
   - If yes, please upload (file type “Other”) the original approval document you received from your ethics committee. If the original document is in another language, please also provide an English translation.

_O_ Uploaded ___ N/A

**Yes I obtain ethics approval in our institution (CR323312)**

1. If you prospectively recruited human participants for the study – for example, you conducted a clinical trial, distributed questionnaires, or obtained tissues, data or samples for the purposes of this study, please report in the Methods:
   1. the day, month and year of the **start and end** of the recruitment period for this study.
   2. whether participants provided informed consent, and if so, what type was obtained (for instance, written or verbal, and if verbal, how it was documented and witnessed). If your study included minors, state whether you obtained consent from parents or guardians. If the need for consent was waived by the ethics committee, please include this information.

___ Completed _O_ N/A

1. If you are reporting a retrospective study of medical records or archived samples, please report in the Methods section:
2. the day, month and year when the data were accessed for research purposes
3. whether authors had access to information that could identify individual participants during or after data collection

_O_ Completed ___ N/A
 **This study analyzed the Korea National Health and Nutrition Examination Survey (KNHANES) to confirm the association between liver profiles and insulin resistance. The KNHANES is a cross-section designed and Korea-nationwide dataset annually conducted by the Korea Centers for Disease Control and Prevention (KCDC). All of the participants in the 2011–2019 KNHANES signed an informed consent form. All data were accessed in compliance with the Helsinki Declaration. The individual approval of the Institutional Review Board of Wonju Severance Christian Hospital was waived since the KNHANES data are publicly available and all subjects in these surveys are fully anonymized and un-identified (IRB number: CR323312). In detail, the dataset was compiled from the KNHANES official website (https://knhanes.kdca.go.kr/knhanes/eng/index.do) after database access permission was granted.**
