# Supplementary material for: Effectiveness of the ALT/AST ratio for predicting insulin resistance in a Korean population: A large-scale, cross-sectional cohort study
Source: PLoS One. 2024 May 17;19(5):e0303333. doi: 10.1371/journal.pone.0303333 (PMC11101110; doi:10.1371/journal.pone.0303333)
Supplement: S1 File — (ZIP) [file pone.0303333.s002.zip › PLOSone_Figure.docx]

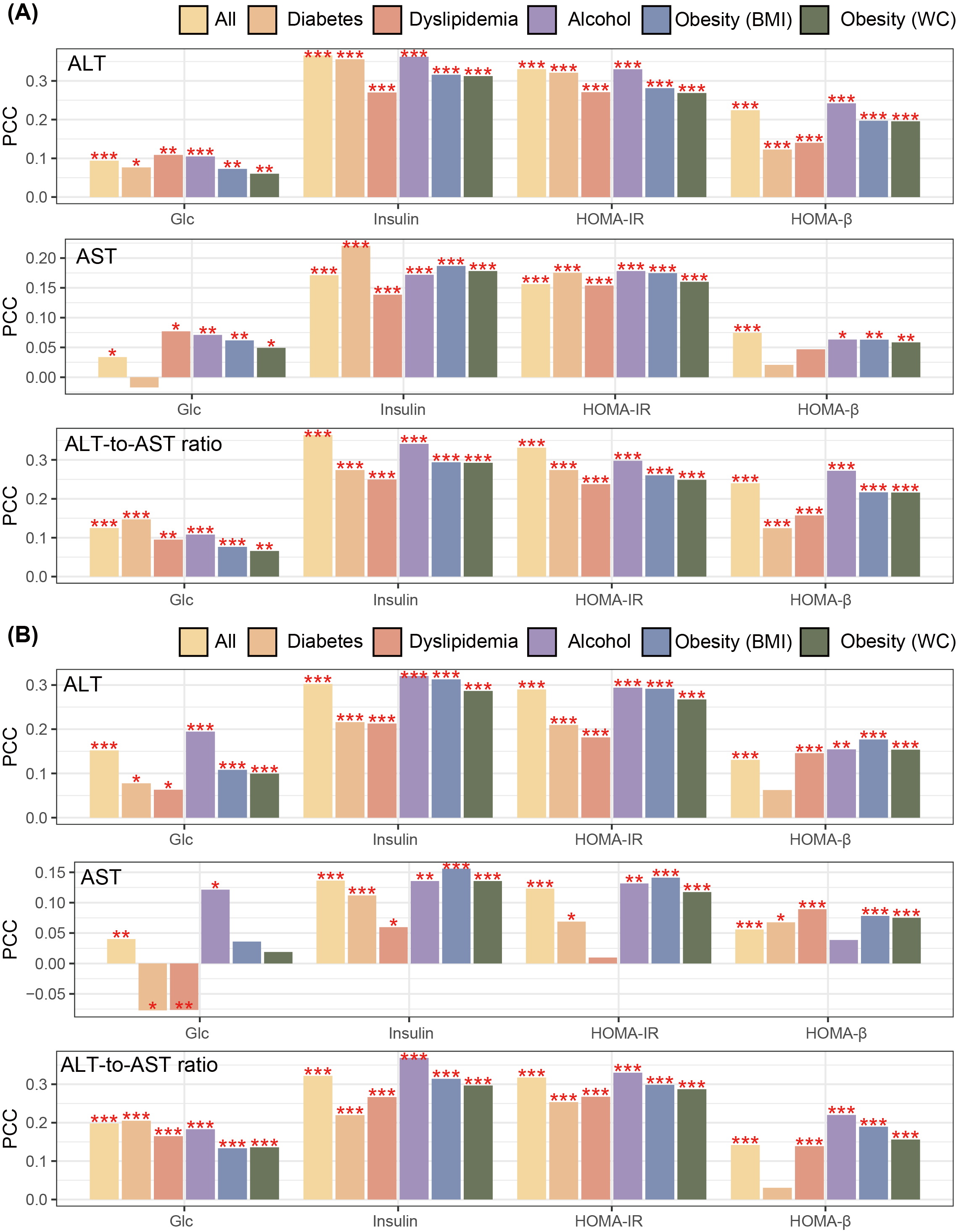


**Figure 1.** Association of liver profiles and IR indices. *, **, and *** denote *p*-value < 0.05, *p* < 0.01, and *p* < 0.001 that were calculated by Peason’s correlation method, respectively. Abbreviations: PCC, Pearson’s correlation coefficient; ALT, alanine aminotransferase; AST, aspartate aminotransferase; HOMA-IR, Homeostasis model assessment for insulin resistance; HOMA-β, HOMA for β cell.


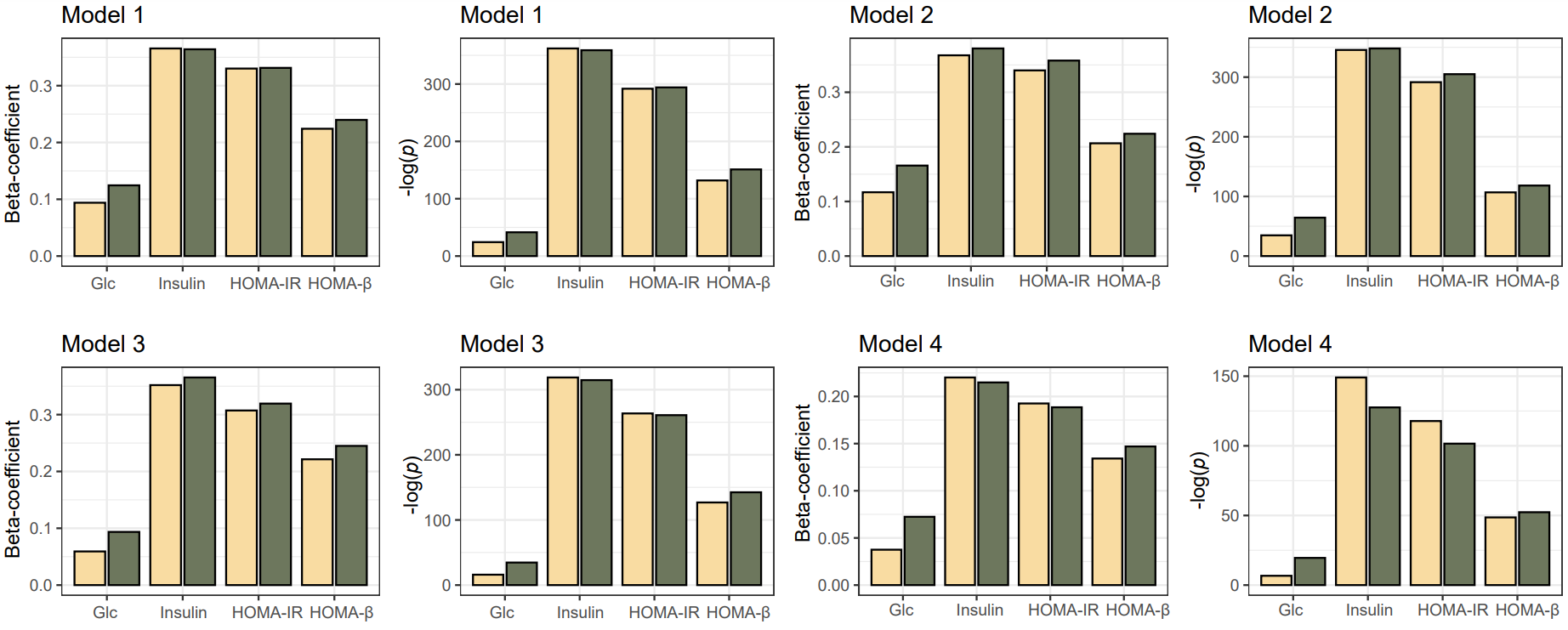


**Figure 2.** Comparative analysis of the degree of association of ALT and ALT-to-AST ratio with IR indices in Korean men. The associational results between liver profile and IR index were calculated four linear regression models including different combinations of covariates. Model 1 is univariate model with setting liver profile and IR indices as dependent and independent variables, respectively. Model 2 includes age as covariates. Model 3 consists of age, diabetes, dyslipidemia, and alcohol consumption as confounders. Model 4 composes age, diabetes, dyslipidemia, alcohol, BMI, and WC as confounder factors. Abbreviation: ALT, alanine aminotransferase; AST, aspartate aminotransferase; HTN; hypertension, ALM, anti-lipidemic medication; BMI, body mass index; WC, waist circumferene; Glc, fasting blood glucose; HOMA-IR, Homeostasis model assessment for insulin resistance; HOMA-β, HOMA for β cell.


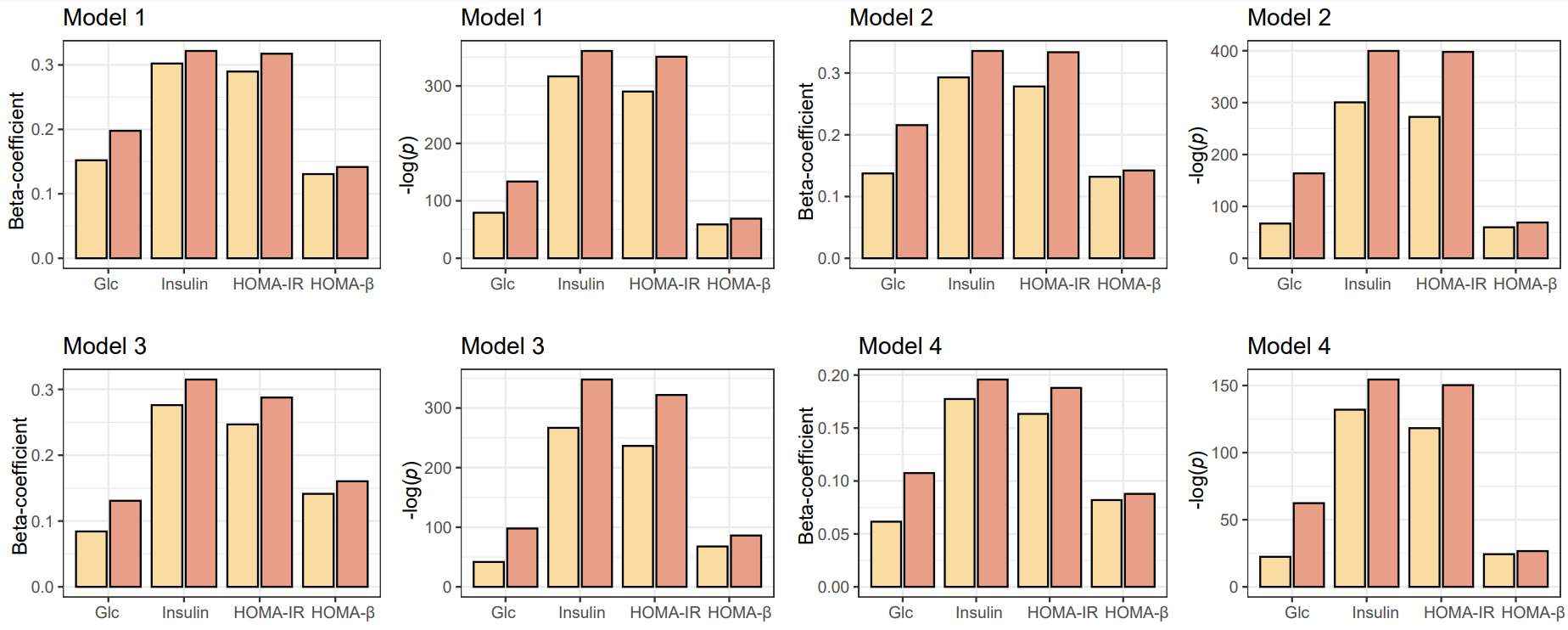


**Figure 3.** Comparative analysis of the degree of association of ALT and ALT-to-AST ratio with IR indices in Korean women. The associational results between liver profile and IR index were calculated four linear regression models including different combinations of covariates. Model 1 is univariate model with setting liver profile and IR indices as dependent and independent variables, respectively. Model 2 includes age as covariates. Model 3 consists of age, diabetes, dyslipidemia, and alcohol consumption as confounders. Model 4 composes age, diabetes, dyslipidemia, alcohol, BMI, and WC as confounder factors. Abbreviation: ALT, alanine aminotransferase; AST, aspartate aminotransferase; HTN; hypertension, ALM, anti-lipidemic medication; BMI, body mass index; WC, waist circumferene; Glc, fasting blood glucose; HOMA-IR, Homeostasis model assessment for insulin resistance; HOMA-β, HOMA for β cell.


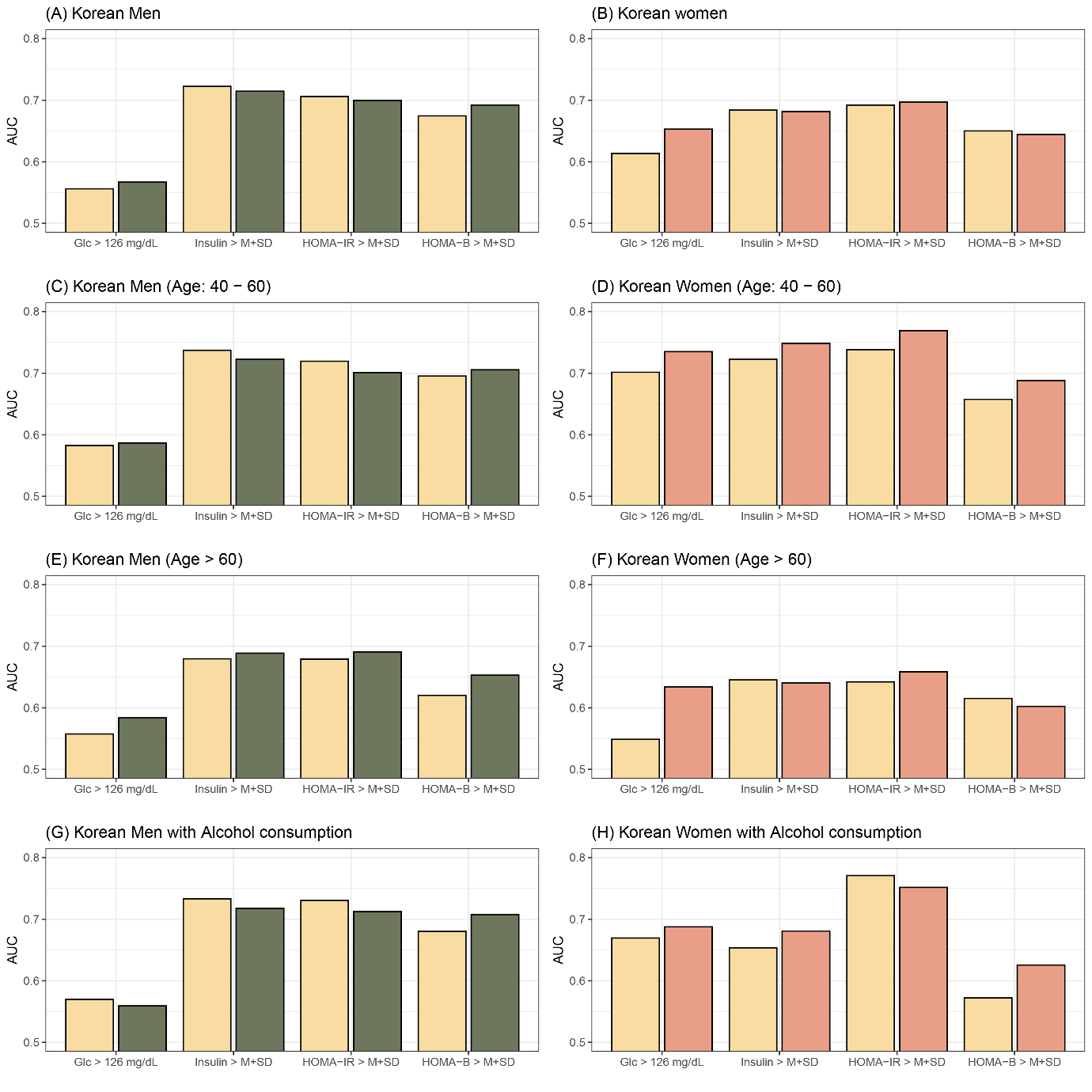


**Figure 4.** Classification performance of ALT and ALT-to-AST ratio for IR status. Abbreviations: Glc, fasting blood glucose; HOMA-IR, Homeostasis model assessment for insulin resistance; HOMA-β, HOMA for β cell.
