# Supplementary figures and images for: Effectiveness of the ALT/AST ratio for predicting insulin resistance in a Korean population: A large-scale, cross-sectional cohort study
Source: PLoS One. 2024 May 17;19(5):e0303333. doi: 10.1371/journal.pone.0303333 (PMC11101110; doi:10.1371/journal.pone.0303333)

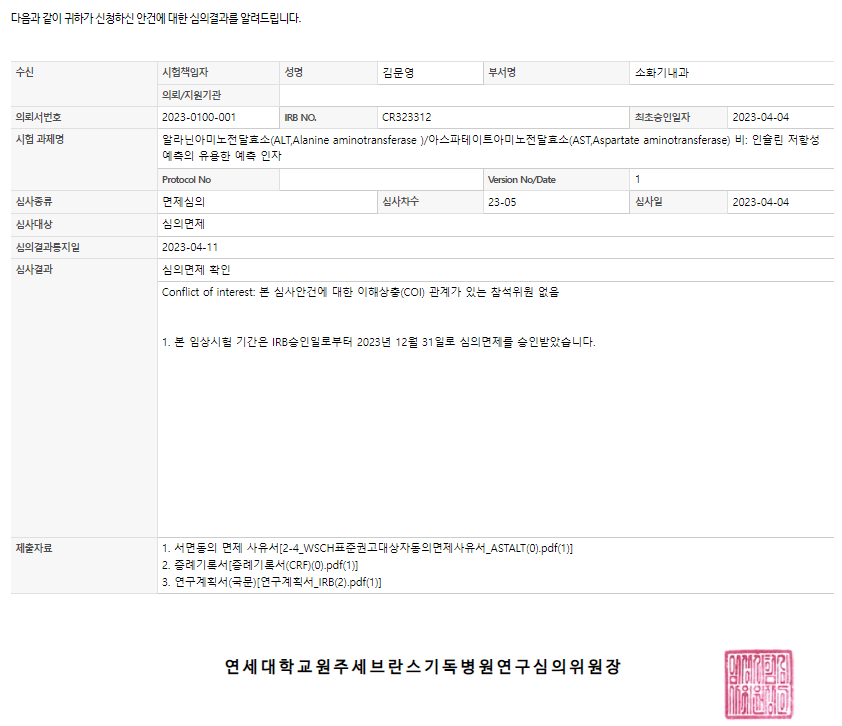

Supplement: S1 File — (ZIP) [file pone.0303333.s002.zip › PLOSone_IRB result.png]
