# Supplementary material for: Effectiveness of the ALT/AST ratio for predicting insulin resistance in a Korean population: A large-scale, cross-sectional cohort study
Source: PLoS One. 2024 May 17;19(5):e0303333. doi: 10.1371/journal.pone.0303333 (PMC11101110; doi:10.1371/journal.pone.0303333)
Supplement: Attachment — Submitted filename: renamed_221ba.docx [file pone.0303333.s003.docx]

**Reviewer report**

Seul ki han et all. investigated that the AST/ALT ratio is a useful marker for insulin resistance. I suggest that the study be organised in line with the reports I have mentioned.

1. Recommendation (Answer options: Major Revision)
2. Is the manuscript technically sound, and do the data support the conclusions? (Answer options: Partly)
3. Has the statistical analysis been performed appropriately and rigorously? (Answer options: N/A)
4. Does the manuscript adhere to the PLOS Data Policy? Additional details can be found at http://journals.plos.org/plosone/s/materials-and-software-sharing. (Answer options: Yes)
5. Is the manuscript presented in an intelligible fashion and written in standard English? (Answer options: Yes)
6. Review Comments to the Author (minimum 200 characters)
7. PLOS authors have the option to publish the peer review history of their article (https://journals.plos.org/plosone/s/editorial-and-peer-review-process#loc-peer-review-history). If published, this will include your full peer review and any attached files. Do you want your identity to be public for this peer review? (Answer options: No)
7a. If you answered "Yes" above, that you would like your identity to be public, please provide your full name, as it should appear on the published peer review. Please do not sign the review on behalf of another person.

**Confidential to Editor:**
1. Do you have any potentially competing interests? None
2. Did you receive any assistance in preparing this review (e.g. from a post-doc or graduate student)? If yes, please provide their name and title. If you have chosen to reveal your identity to the authors in question 7 above, please include the name of the person who assisted you in the response to question 7 as well. (optional)
3. Do you want to get recognition for this review on Web of Science (https://clarivate.com/products/scientific-and-academic-research/research-discovery-and-workflow-solutions/researcher-profiles/)? If you opt in, your Web of Science profile will automatically be updated to show a verified record of this review in full compliance with the journal's review policy. If you don't have a Web of Science profile, you will need to first create a free account. (Answer options: No)

1-**İn the Method/data set and participants**

Last sentence ^^ Finally, we selected a population of 11,547 individuals (men: 5,001, women: 6,546) to determine the association between ALT/AST ratio and NAFLD.

However, to determine the association of ALT/AST ratio with NAFLD, there must be a definite indication of the presence of NAFLD in the participants, such as abdominal ultrasonography or liver biopsy, and the association must be analysed accordingly. Otherwise, such a result cannot be reported.

2-While the relationship with diabetes, dyslipidaemia and obesity is defined in **Figure 1**, was the relationship with the healthy control group analysed?

2-**İn the Table 1:** ALT/AST ratio analysed according to tertile groups; the definition of tertile groups should be better explained.

3-**İn the Table 1**: ALT/AST ratio analysed according to tertile groups; tertile groups should be better explained. 3-Table 2: ALT/AST ratio analysed by tertile groups; tertile groups need to be better explained. This situation prevents the evaluation of the relationship between ALT/AST ratio and insulin resistance independently of liver diseases.

4- There are many studies investigating the relationship between ALT/AST ratio and insulin resistance in obese, healthy individuals, NAFLD, etc. in the literature. At this point, I recommend hypothesising by carefully reading the literature in the selection of method material.
